# Comparison of MRI vs. [^18^F]FDG PET/CT for Treatment Response Evaluation of Primary Breast Cancer after Neoadjuvant Chemotherapy: Literature Review and Future Perspectives

**DOI:** 10.3390/jcm12165355

**Published:** 2023-08-17

**Authors:** Matteo Caracciolo, Angelo Castello, Luca Urso, Francesca Borgia, Maria Cristina Marzola, Licia Uccelli, Corrado Cittanti, Mirco Bartolomei, Massimo Castellani, Egesta Lopci

**Affiliations:** 1 Nuclear Medicine Unit, Oncological Medical and Specialists Department, University Hospital of Ferrara, 44124 Ferrara, Italy; 2 Nuclear Medicine Unit, Fondazione IRCCS Ca’ Granda, Ospedale Maggiore Policlinico, 20122 Milan, Italy; 3 Department of Nuclear Medicine PET/CT Centre, S. Maria della Misericordia Hospital, 45100 Rovigo, Italy; 4 Department of Translational Medicine, University of Ferrara, 44121 Ferrara, Italy; 5 Nuclear Medicine Unit, IRCCS—Humanitas Research Hospital, Via Manzoni 56, 20089 Rozzano, Italy

**Keywords:** breast cancer, FDG, PET/CT, MRI, neoadjuvant chemotherapy, response, pathological complete response

## Abstract

The purpose of this systematic review was to investigate the diagnostic accuracy of [^18^F]FDG PET/CT and breast MRI for primary breast cancer (BC) response assessment after neoadjuvant chemotherapy (NAC) and to evaluate future perspectives in this setting. We performed a critical review using three bibliographic databases (i.e., PubMed, Scopus, and Web of Science) for articles published up to the 6 June 2023, starting from 2012. The Quality Assessment of Diagnosis Accuracy Study (QUADAS-2) tool was adopted to evaluate the risk of bias. A total of 76 studies were identified and screened, while 14 articles were included in our systematic review after a full-text assessment. The total number of patients included was 842. Eight out of fourteen studies (57.1%) were prospective, while all except one study were conducted in a single center. In the majority of the included studies (71.4%), 3.0 Tesla (T) MRI scans were adopted. Three out of fourteen studies (21.4%) used both 1.5 and 3.0 T MRI and only two used 1.5 T. [^18^F]FDG was the radiotracer used in every study included. All patients accepted surgical treatment after NAC and each study used pathological complete response (pCR) as the reference standard. Some of the studies have demonstrated the superiority of [^18^F]FDG PET/CT, while others proved that MRI was superior to PET/CT. Recent studies indicate that PET/CT has a better specificity, while MRI has a superior sensitivity for assessing pCR in BC patients after NAC. The complementary value of the combined use of these modalities represents probably the most important tool to improve diagnostic performance in this setting. Overall, larger prospective studies, possibly randomized, are needed, hopefully evaluating PET/MR and allowing for new tools, such as radiomic parameters, to find a proper place in the setting of BC patients undergoing NAC.

## 1. Introduction

Breast cancer (BC) is the most common cancer in the world, accounting for at least 30% of female neoplasms and with an increasing incidence of approximately 0.3% per year since 2004 [1]. Neoadjuvant chemotherapy (NAC) is the first-line treatment option in case of non-operable and/or locally advanced BC and should start as soon as diagnosis and staging are completed (ideally within 2–4 weeks) [2,3,4]. This strategy leads to a downstage of the primary tumor, allowing a considerable number of patients to undergo breast-conserving surgery, converting mastectomy to quadrantectomy. Moreover, a reduced need for axillary lymph node dissection is reported after NAC, with a consequent reduced surgical morbidity [5,6]. Several literature reports agree that pathological complete response (pCR) is the best tool for the evaluation of tumor response after NAC, as it has been demonstrated to be a strong prognostic factor [7,8,9]. In this setting, early assessment of the response after NAC is of paramount importance in order to verify the therapy’s effectiveness, identify non-responding patients, and guide the selection of an alternative treatment option [10]. Through comparison of clinical breast examinations, such as mammography, ultrasound (US), and magnetic resonance imaging (MRI), it has been found that the latter is the most accurate tool for assessing tumor response and residual tumor after NAC, but there are still some important issues that should be addressed [11]. In fact, based mostly on anatomical variations, MRIs have shown high specificity (83–91%) and moderate sensitivity (63–75%) [12]. These variations can be the results of, for example, fibrosis, tumor fragmentation, or anti-angiogenic effects leading to an under or overestimation of the response. Furthermore, MRI features have different predictive values across the various BC subtypes, and this does not allow the evaluation of possible distant metastasis [13]. In the last few years, the use of [^18^F]fluorodeoxyglucose (FDG) positron emission tomography/computed tomography (PET/CT) has been investigated in this scenario with encouraging preliminary results showing a significant correlation between pCR and longer survival in patients with a complete metabolic response on [^18^F]FDG PET/CT, which could overcome some of the above-mentioned limitations [14]. The aim of this systematic review was to investigate the diagnostic accuracy of [^18^F]FDG PET/CT and MRI for response assessment after NAC in BC patients and to evaluate future perspectives in this setting.

## 2. Materials and Methods

Our systematic review was conducted following the “Preferred Reporting Items for a Systematic Review and Meta-Analysis” (PRISMA) guidelines [15].

### 2.1. Literature Search Strategy and Selection of the Studies

A comprehensive search of the literature was conducted through three bibliographic databases (i.e., PubMed, Scopus, and Web of Science) for papers published up to 6 June 2023, with a starting date limit set to 2012. The search keywords included: ((((locally advanced breast cancer [Text Word]) OR (breast cancer[Text Word])) AND (neoadjuvant chemotherapy[Text Word])) AND (((MRI[Text Word]) OR (magnetic resonance imaging[Text Word])) OR (MR[Text Word]))) AND ((PET[Text Word]) OR (positron emission tomography[Text Word])). Additionally, the references of the articles as well as unpublished and ongoing studies in the ClinicalTrials.gov database were also independently searched by two authors (M.C. and A.C.). Full texts were retrieved when the title and abstract were considered relevant, whereas disagreements were solved by a consensus including a third author (E.L.). The inclusion criteria were as follows: histology-proven breast cancer; MRI and PET/CT performed after NAC; post-surgery pathologic response as the gold standard. Exclusion criteria for our systematic review were: non-English language, studies with animal models, case reports/poster presentations/letters in the topic of interest, small series (i.e., less than 10 patients), published more than ten years ago, involving hybrid imaging only, or with other non-FDG radiotracers. 

### 2.2. Data Collection and Extraction

The three above-mentioned reviewers (M.C., A.C. and E.L.) independently carried out the data collection process in order to reduce possible bias.

For each of the selected studies in our review, the data extracted were general study information (i.e., authors, publication year, study design, number of institutions included, funding sources, and country), patients’ features (i.e., number of cohorts, age, BC histological features), imaging performed, and response assessment parameters.

### 2.3. Quality Assessment

To assess the risk of bias in individual studies as well as concerns regarding the applicability of review questions, the Quality Assessment of Diagnosis Accuracy Study (QUADAS-2) method was adopted. Four domains, patient selection, index test, reference standard, and flow and timing, were evaluated for the risk of bias. Three domains (i.e., patient selection, index test, and reference standard) were investigated in terms of concerns regarding applicability [16]. 

## 3. Results

### 3.1. Literature Search 

**Figure 1:**
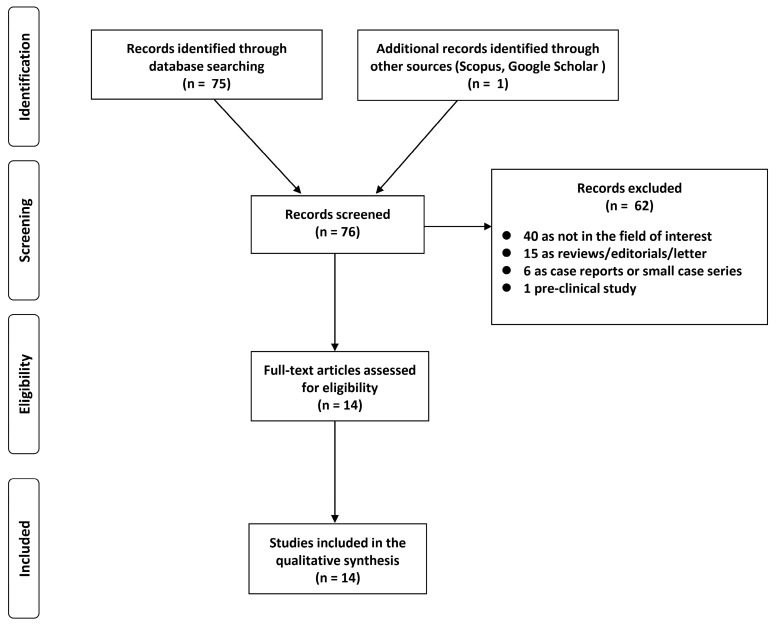
PRISMA flowchart of the study.

A total of 76 studies were identified and screened. Considering predefined eligibility criteria, out of these 76 articles assessed for eligibility, 62 records were excluded (40 as not in the field of interest; 15 as reviews, editorials, or letters; 6 as case reports; 1 preclinical study). After full-text examination, the remaining 14 articles were suitable for inclusion in our systematic review (Figure 1) [17,18,19,20,21,22,23,24,25,26,27,28,29,30].

### 3.2. Basic Characteristics 

Overall, 842 was the total number of included patients, ranging between 11 and 188 per study. Four studies (35.7%) enrolled more than 50 patients. Eight out of fourteen studies (57.1%) were prospective, while all except one study were conducted in a single center. Characteristics of the included studies are summarized in Table 1.

### 3.3. Imaging and Technical Aspects

In most of the included studies (71.4%), MRI scans were acquired on a 3.0 Tesla (T) system with a dedicated breast coil. Three out of fourteen studies (21.4%) used both 1.5 and 3.0 T MRI and only two used 1.5 T. One study also included contrast-enhanced US in the comparison of techniques and Tokuda et al. evaluated dedicated-breast PET (dbPET) [22,28]. [^18^F]FDG was the radiotracer used in every study included; PET data were acquired in a two-dimensional mode after na on-contrast CT scan from the base of the skull to the pelvis. All patients received surgical treatment after NAC and each study compared the diagnostic value of MRI and PET/CT, considering pCR as the reference standard. Core-needle biopsies of the lesion were executed before NAC and more tumor samples were obtained after surgery; all specimens were analyzed by an experienced breast pathologist blinded to the imaging results. Regarding response assessment, parameters used were as follows: Response Evaluation Criteria in Solid Tumors (RECIST), PET Response Criteria in Solid Tumors (PERCIST), the percentage change of MR parameters such as largest tumor diameter (LD), unidimensional diameter (1D), tumor volume (TV), and the percentage variation of PET parameters such as standardized uptake value (SUV), standardized uptake value corrected for lean body mass (SUL), and metabolic tumor volume (MTV). Detailed information is reported in Table 2.

### 3.4. Main Findings 

In the last decade, several studies have compared different imaging methods in the evaluation of the response to NAC in patients with BC. Some of these showed a better performance for [^18^F]FDG PET/CT in this patient setting [17,18,21]. Tateishi et al. [17] reported for the first time the diagnostic accuracy of percentage variation (∆) of maximum standardized uptake (SUVmax) in predicting pCR after NAC compared with the kinetic parameters obtained from dynamic contrast-enhanced (DCE) MRI images. In their cohort, [^18^F]FDG PET/CT was superior to MRI for the prediction of pCR (∆SUVmax (90.1%) vs. ∆kinetic (83.8%) or ∆AUC90 (76.8%), *p* < 0.05). Moreover, Pahk et al. [26] evaluated the effectiveness of interim PET/CT (i.e., a mid-point scan after the third or the fourth cycle of therapy) for predicting pCR in a group of Luminal-B histotypes. ∆SUVmax of the pCR subgroup was significantly higher than the non-pCR group (*p* < 0.001); a cut-off of ∆SUV of 69% was proposed for discriminating pCR from non-pCR patients after receiver-operating characteristic (ROC) analysis (*p* < 0.0001). Conversely, no statistically significant difference in size change between pCR and non-pCR was found in MRI data. Moreover, the area under the curve (AUC) of [^18^F]FDG PET/CT was significantly higher than that of MRI (0.9 vs. 0.65), demonstrating that [^18^F]FDG PET/CT could be more accurate than MRI (*p* = 0.04). More recently, in a study by Tokuda et al. [21], the performance of whole-body PET and DCE-MRI was compared with dbPET, a recently introduced high-resolution imaging acquired on hanging uncompressed breast, using a full-ring breast-dedicated tomograph [31]. The sensitivity, specificity, and AUC for predicting pCR on dbPET were 85.7%, 72.7%, and 0.818, respectively, while those for whole-body PET were 71.4%, 77.3%, and 0.727, respectively, and those for MRI were 100, 50, and 0.773, respectively. Together, these results suggest that dbPET was the best predictor of pCR after NAC. 

Innovative results have also been obtained from Cho et al. [29], with the first prospective study comparing the performances of single-voxel proton magnetic resonance spectroscopy (MRS) and [^18^F]FDG PET/CT in predicting the pathological residual tumors in 35 patients who received NAC. Changes in SUVmax, peak standardized uptake (SUVpeak), total lesion glycolysis (TLG) from PET/CT, and total choline-containing compounds by MRS were measured. Mean percentages reductions of all these parameters were higher in the pCR group than in the non-pCR group (MRS −80.3 ± 13.9% vs. −32.1 ± 49.4%, *p* = 0.025; SUVmax −54.7 ± 22.1% vs. −26.3 ± 33.7%, *p* = 0.058; SUVpeak −60.7 ± 18.3% vs. −32.3 ± 23.3%, *p* = 0.009; TLG −89.5± 8.5% vs. −52.6 ± 36.2%, *p* = 0.020), demonstrating a comparable performance between the two techniques in prediction of pCR. Another interesting aspect of this study is that the AUC value of TLG (0.879) was similar to those of SUVpeak (0.862) and SUVmax (0.822), highlighting a possible use of this parameter. 

Conversely, other important studies have shown the superiority of MRI in predicting the pCR in this scenario. Kim et al. [24] compared ∆SUVmax with the volume reduction rate by three-dimensional MRI: the volume reduction of primary BC reported by MRI demonstrates the highest correlation with histopathological tumor regression (*p* < 0.0001). Volume reduction rate demonstrated the largest value after ROC analysis (AUC = 0.9), followed by SUVmax decrease (AUC = 0.875) and diameter decrease rate (AUC = 0.849). 

In a recent study, Choi et al. [30] evaluated the values of [^18^F]FDG PET/CT and MRI for response assessment in thirty-three patients before and one to four weeks after NAC. Following NAC, they found significant differences between responders and non-responders in terms of hottest voxel (SULpeak: 0.9 ± 0.4 vs. 2.4 ± 1.7; *p* < 0.001), metabolic tumor volume (MTV: 0.1 ± 0.1 cm^3^ vs. 12.0 ± 35.5 cm^3^; *p* < 0.001) for PET/CT, and unidimensional diameter (ID: 2.5 ± 1.4 cm vs. 4.7 ± 3.0 cm; *p* = 0.0003) and tumor volume (TV: 5.02 ± 5.73 cm^3^ vs. 31.3V46.0 cm^3^; *p* = 0.038) for MRI values. However, sensitivity, specificity, accuracy, positive predictive value (PPV), and negative predictive value (NPV) of the pathological response with PET/CT and MRI were 100%, 25%, 63.6%, 58.6%, and 100%, and 88.2%, 62.5%, 75.7%, 71.4%, and 83.3%, respectively. Therefore, [^18^F]FDG PET/CT showed lower specificity and accuracy, but higher sensitivity than MRI, although no significant difference was found between the two methods.

Therefore, probably due to these discordant results, other recent studies have focused on the complementary value of MRI and PET/CT. Park S.H. et al. [18] aimed to compare the use of diffusion-weighted (DWI) MRI and PET/CT to predict pCR in a cohort of 34 patients. The best cut-off values for differentiating pCR from non-pCR were a 54.9% increase in apparent diffusion coefficient after chemotherapy and a 63.9% decrease for SUVmax. Using these values, DWI showed 100% sensitivity and 70.4% specificity and PET/CT showed 100% sensitivity and 77.8% specificity. There was a trend toward improved specificity and accuracy with the combined use of DWI and PET/CT compared with DWI alone (*p* = 0.063 for both). Indeed, the combination of MRI and PET/CT increased the diagnostic selectivity to 88.9%. To the best of our knowledge, Kitajima et al. [20] performed the first direct comparison of RECIST 1.1 and PERCIST 1.0 for predicting the pathological response to NAC. A significant difference was observed between RECIST 1.1 and PERCIST 1.0 (k = 0.103, *p* < 0.0001) for response classification: tumor response was downgraded in 2 patients (6.2%) and upgraded in 23 cases (71.9%) using PERCIST 1.0. Moreover, sensitivity and specificity to predict pCR were significantly different between the classification: 8.6% and 94% with RECIST 1.1 and 100% and 22.2% with PERCIST 1.0, respectively (*p* = 0.000444, *p* = 0.00087), hinting at a complementary function of the two different imaging methods. 

In addition, some papers suggested a difference in efficacy between PET/CT and MRI depending on the BC histotypes. For example, Schmitz et al. [19] explored the use of MRI and [^18^F]FDG PET/CT in monitoring primary tumor response to NAC in patients affected by different BC subtypes. In a cohort of 188 patients, differences in efficacy regarding human epidermal growth factor receptor 2 (HER2)-positive, estrogen receptor (ER)-positive, and triple-negative tumors were analyzed. For HER2-positive (46 patients), MRI resulted in the strongest predictor (AUC: 0.735; sensitivity 36.2%), outperforming PET/CT (AUC: 0.543; *p* = 0.04), and with comparable results to combined imaging (AUC: 0.708; *p* = 0.213). For ER-positive cases (87 patients), the combination of MRI and PET/CT was slightly superior (AUC: 0.818; sensitivity 55.8%) than MRI alone (AUC: 0.742; *p* = 0.117) and PET/CT alone (AUC: 0.791). However, even though relatively large numbers of ER-positive patients were included, no significant differences were found. Regarding triple-negative (55 patients), MRI (AUC: 0.855; sensitivity 45.4%), PET/CT (AUC: 0.844; *p* = 0.220), and combined imaging (AUC: 0.868; *p* = 0.213) produced comparable results.

Very recently, Baysal and colleagues [22] evaluated the agreement between MRI and PET/CT response in 88 BC patients who underwent surgery following NAC. Tumor diameters and SUVmax were significantly decreased (*p* < 0.001), with MRI being more sensitive in ER-positive and E-cadherin-negative patients, while PET/CT was more sensitive in those with HER-2 overexpression, Luminal-B, or proliferation rate >14% (*p* = 0.01). Selectivity, sensitivity, PPV, and NPV for MRI were 80.7%, 65.2%, 75%, and 72.4%, respectively; on the other hand, the same parameters for PET/CT were 75.7%, 100%, 57.9%, and 100%, respectively. 

Table 3 details the diagnostic performance from the above-mentioned studies to predict pCR.

### 3.5. Risk of Bias Evaluation

The QUADAS-2 quality assessment (Table 4) was used to assess the risk of bias. All studies used post-surgery pathologic results as the gold standard. Overall, results show that the quality of the included articles was satisfactory with moderately low concern.

## 4. Discussion

The introduction of NAC has recently acquired an important role in the treatment of locally advanced BC, allowing high percentages of tumor downstaging and facilitating surgery conversion to less aggressive approaches [32]. It has been reported that [^18^F]FDG PET/CT and MRI are the most accurate tools for predicting pCR, outperforming both US and mammography [33]. Innovative tools such as DWI- and DCE-MRI overcome digital mammography in terms of evaluation of tissue changes and intra-tumoral variations, allowing a more accurate assessment of lesion response after NAC [34]. Moreover, the American College of Radiology Imaging Network trial recently compared clinical evaluation and mammography to MRI, showing that MRI had the best accuracy for detecting pCR. In particular, the longest diameter by MRI had a better accuracy both in single and multiple masses as well as in tumors without ductal carcinoma in situ in comparison to mammography [35]. Despite this evidence, according to some studies, residual disease may be overestimated or underestimated. Causes of overestimation could be, for example, fibrosis or post-treatment inflammatory processes mimicking residual disease. Moreover, fibroadenomas and other benign findings may decrease or remain stable and be mistaken for residual disease [36]. Instead, an underestimation may be due to tumors with non-mass morphology or non-concentric shrinkage patterns, or suppressed enhancement caused by antiangiogenic therapy [37]. Lastly, some studies have pointed out that the sensitivity of post-NAC MRI to detect persistent lymph node metastasis is moderate, ranging between 61 and 72%. Putting together this information, it appears clear the need for MRI improvement or new tools to solve these problems [38]. An interesting possibility has recently been explored by a study by Hayashi et al., which highlighted the utility of a second-look US after MRI to predict pCR; in a large cohort of 1274 patients, the PPV was greatest combined with the two methods versus MRI alone (86.8% vs. 79.4%), particularly in the ER-/HER2+ tumors (98.1%), although it remained difficult to identify the residual in situ disease using conventional radiology due to the morphological and biological variations, and it is also not easy to clearly evaluate its accuracy through clinical trials in terms of objectivity and reproducibility [39].

**Figure 2:**
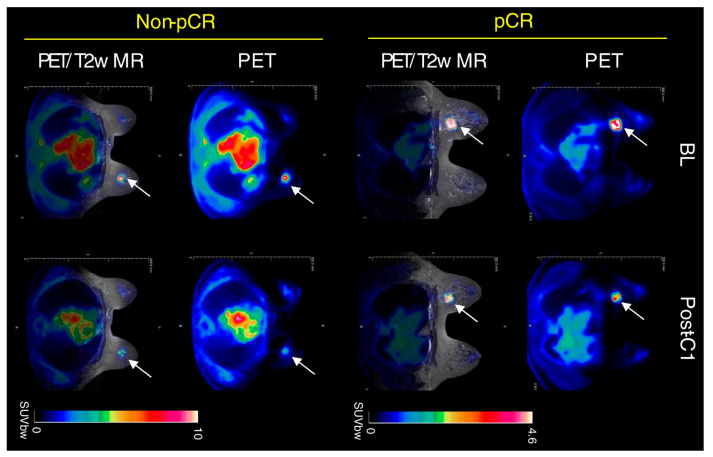
Clinical PET/MR images of response to therapy in triple-negative BC after NAC. A tumor is indicated by a white arrow. Adapted from Roy S et al. [44] published under a Creative Commons Attribution 4.0 International License http://creativecommons.org/licenses/by/4.0/, accessed on 27 June 2023.

Nuclear medicine offers a viable alternative to overcome these problems for the evaluation of tumor residual after NAC. In a meta-analysis of 19 studies, the sensitivity, specificity, PPV, NPV, and diagnostic odds ratio of [^18^F]FDG PET/CT to predict pCR in primary BC were 84%, 66%, 50%, 91%, and 11.90, respectively [40]. More recently, Aydin et al. [41] analyzed PET/CT results in 186 patients before and after the completion of NAC. Of note, the sensitivity, specificity, PPV, and NPV of [^18^F]FDG PET/CT to determine pCR were 100%, 72.2%, 72.5%, and 100%, respectively, confirming that PET/CT is a useful tool in this subgroup of patients. Nevertheless, [^18^F]FDG PET/CT certainly has some limitations compared to MRI; for example, the anatomical resolution is lower, and generally, the cost is higher, leading to a problem of cost-effectiveness. Despite this evidence, only a few studies have focused on the direct comparison between the two scans, of which, to the best of our knowledge, the review by Li et al. [42] is the only recent comparing study relative to the diagnostic performance of MRI and PET/CT after NAC. In particular, the pooled sensitivity and specificity of MRI were 0.88 and 0.69, respectively, whereas for PET/CT they were 0.77 and 0.78, respectively. The AUC for MRI and PET/CT were 0.88 and 0.84, respectively. Essentially, MRI showed a better sensitivity and PET/CT a higher specificity in this setting, suggesting a complementarity between the two techniques. Nevertheless, most studies are focused on comparison rather than the assessment of the combined value. To overcome this problem, in recent years important technological advances integrate PET detectors into MRI scanners, creating new PET/MRI hybrid systems that are able to combine metabolic data from PET with anatomic and functional details from MRI (Figure 2) [43].

Sekine et al. evaluated the utility of PET/MRI in predicting pCR in a cohort of 74 patients, with the sensitivity and specificity of PET/MRI being 72.2% and 78.6%, respectively. In particular, they found that the sensitivity of PET/MRI in HER2-positive tumors and the specificity in HER2-negative lesions were excellent, meaning that tumor disappearance was well identified in HER2-positive cases, while the residual disease was easily detected in HER2-negative cases [45]. More recently, de Mooij et al. suggested that the diagnostic performance in predicting primary tumor response can be improved with quantitative [^18^F]FDG PET/MR imaging variables; the complementary values are mainly established by combining the percentage decrease in signal enhancement ratio and SUVmax halfway through NAC, which improved specificity and PPV [46]. These aspects should also be addressed in more prospective multi-institutional studies in order to reduce radiation exposure compared to conventional staging scans and to develop a tailored approach to therapy as well as pretreatment patient stratification.

These results are encouraging, but in order to further increase diagnostic accuracy, nuclear medicine can offer valid alternatives, such as non-FDG radiotracers, the use of volumetric parameters, or the introduction of radiomics parameters. In fact, new molecules labeled other than [^18^F]FDG could be useful to predict response to NAC, analyzing aspects beyond glucose metabolism, in particular, the use of some radiopharmaceuticals in relation to tumor histotypes: [^18^F]-fluoro-17β-estradiol PET/CT in monitoring ER expression, [^18^F]-fluorothymidine for measurement cell proliferation, or [^18^F]-fluoromisonidazolethe for the evaluation of tumor-related hypoxia [47]. More recently, there are also many expectations regarding fibroblast activation protein (FAP), a molecule overexpressed in the stroma of a variety of cancers, considered a promising target structure for diagnostic and therapeutic approaches [48]. Regarding NAC response assessment, Backhous and colleagues presented initial results using [^68^Ga]-labeled FAP inhibitor (FAPI) PET/MRI in 13 women: the mean breast-tumor-to-background ratio was 0.9 for pCR and 2.1 for non-pCR (*p* = 0.001). Integrated PET/MRI could classify breast response correctly in all 13 women based both on readers’ visual assessment and the tumor-to-background ratio, with a diagnostic performance of PET/MRI trending toward a gain over MRI alone, clearly supporting future prospective studies in this field [49].

The use of volumetric parameters extracted from [^18^F]FDG PET/CT is another promising tool to assess response after NAC in BC patients [50]. In particular, Evangelista et al. [51] reported for the first time that baseline TLG could predict disease-free survival. Similarly, Urso et al. [52] reported that the SUVmean of the primary tumor at baseline [^18^F]FDG PET/CT was higher in Luminal-B patients achieving pCR after NAC. Conversely, MTV and TLG of the primary tumor were lower in Luminal-B and HER2-positive patients who obtained a pCR, suggesting that the primary tumor volume could be a key factor in this subgroup of BC patients undergoing NAC. Interestingly, no parameter resulted in a reliable predictor of pCR after NAC in triple-negative BC, although four volumetric parameters (i.e., MTV and TLG from primary tumor as well as from the whole-body load of disease) could discriminate patients dead at follow-up among those with pCR after NAC. This evidence is consistent with several other pieces of evidence from the literature reporting the prognostic relevance of semi-quantitative parameters on [^18^F]FDG PET/CT in different subtypes of BC [53,54,55,56].

Finally, several authors already investigated the potential usefulness of radiomics analysis extracted from baseline [^18^F]FDG PET/CT prior to the start of NAC to predict both pCR and survival [57,58,59]. Despite very promising results, the main limit to the wide use of radiomics in clinical practice is related to the lack of reproducibility and standardization [60]. The training of artificial intelligence systems could represent a way to overcome these issues, although a large amount of data is needed to obtain reliable algorithms [61].

Some limitations of this review need to be pointed out. Firstly, the study did not analyze separately BC with different receptor status and histology subtypes. However, this is an open issue that the currently available literature still cannot solve. It is desirable that future studies focusing on this setting of disease will pay more attention to the histology of BC of their cohorts. Moreover, the number of studies considered was small, with the majority deriving from a single center and some of them being retrospective. In addition, study design, therapy schemes, and patient heterogeneity in our opinion did not allow for performing a significant statistical analysis. Finally, different MRI sequences and PET-CT acquisition tools were compared, which could lead to measurement errors.

## 5. Conclusions

**Figure 3:**
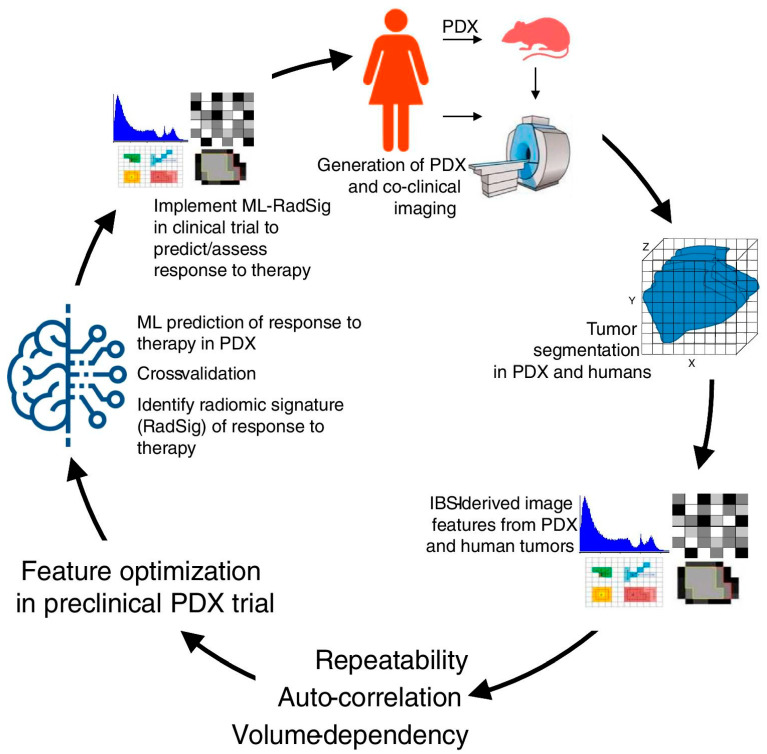
Overview of methodology in co-clinical FDG-PET radiomic signature for predicting response to neoadjuvant chemotherapy in triple-negative breast cancer. Reproduced from Roy S et al. [44] published under a Creative Commons Attribution 4.0 International License http://creativecommons.org/licenses/by/4.0/, accessed on 27 June 2023.

In the present study, we investigated the role of [^18^F]FDG PET/CT in comparison to MRI for the assessment of BC patients undergoing NAC. The data derived from our systematic research prove that part of the literature is in favor of PET/CT and part highlights MRI as superior in this setting. Recent studies indicated that [^18^F]FDG PET/CT has a higher specificity, while MRI has a higher sensitivity in assessing pCR in BC patients after NAC. The complementary value of the combined use of these modalities most likely represents the most important tool we have to improve diagnostic performance in this setting. However, further larger prospective studies, possibly randomized, and evaluating PET/MR and radiomic parameters (Figure 3) are needed.

## Figures and Tables

**Table 1:** General study information.

Authors [Ref.]	Year	Country	Study Design/N° of Involved Centers	Funding Sources
Amioka et al. [28]	2016	Japan	Prospective/monocentric	None
An et al. [27]	2015	South Korea	Retrospective/monocentric	National Research Foundation of Korea
Baysal et al. [22]	2022	Turkey	Retrospective/monocentric	None
Choi et al. [30]	2018	South Korea	Prospective/monocentric	None
Kim et al. [24]	2014	South Korea	Retrospective/monocentric	None
Kitajima et al. [20]	2018	Japan	Retrospective/monocentric	None
Cho et al. [29]	2016	South Korea	Prospective/monocentric	National Research Foundation of Korea
Pahk et al. [26]	2015	South Korea	Retrospective/monocentric	Korea Health Industry Development Institute
Park et al. [18]	2012	South Korea	Retrospective/monocentric	Korea Healthcare Technology R&D Project, Ministry for Health, Welfare & Family Affairs, Innovative Research Institute for Cell Therapy
Pengel et al. [25]	2014	Netherlands	Prospective/monocentric	Project Breast CARE
Schmitz et al. [19]	2017	Netherlands	Prospective/monocentric	Project Breast CARE
Simo et al. [23]	2013	Spain	Prospective/monocentric	Not reported
Tateishi et al. [17]	2012	Japan, USA	Prospective/bicentric	None
Tokuda et al. [21]	2021	Japan	Prospective/monocentric	None

**Table 2:** Key study characteristics.

Authors [Ref.]	Sample Size	Mean/Median Age (Years)	Histology	PET Scanner	Response Assessment	pCR
Amioka et al. [28]	63	53.0 (31–69)	LU (5A, 18B, 11HER2), HER2 (8), TP (21)	whole-body	RECIST 1.1	YES
An et al. [27]	16	51.6 (29–69)	DC (19), LC (1)	whole-body	∆SUVmax, ∆LD	NR
Baysal et al. [22]	88	53.09 ± 12.57	LU (26A, 39B, 9HER2), TP (14)	whole-body	RECIST 1.1, PERCIST 1.0	YES
Choi et al. [30]	33	50.0 ± 10	IDC (28), micropapillary (2), ILC (2), metaplastic (1)	whole-body	∆SULpeak, ∆MTV, ∆1D, ∆TV	YES
Kim et al. [24]	38	47.0 (27–70)	DC (54), LC (1), MUC (1)	whole-body	∆SUVmax	NR
Kitajima et al. [20]	32	52.4 (29–74)	DC (29), LC (1), MUC (2)	whole-body	RECIST 1.1, PERCIST 1.0	YES
Cho et al. [29]	35	49.6 (35–65)	DC (33), LC (2)	whole-body	∆SUVmax, ∆LD	YES
Pahk et al. [26]	21	51 (NR)	DC (21)	whole-body	∆SUVmax	NR
Park et al. [18]	34	44 (27–60)	DC (32), MUC (1), other (1)	whole-body	∆SUVmax	NR
Pengel et al. [25]	93	48 (26–68)	DC (85), LC (7)	whole-body	∆SUVmax	YES
Schmitz et al. [19]	188	47 (25–73)	IDC (167), ILC (18), others (3)	whole-body	∆SUVmax, ∆LD	NR
Simo et al. [23]	30	47 (31–70)	LU (12A, 9B), TN (10), HER2 (10)	whole-body	RECIST 1.1, PERCIST 1.0	NR
Tateishi et al. [17]	142	57 (43–72)	DC (131), LC (11)	whole-body	∆SUVmax, ∆LD	NR
Tokuda et al. [21]	29	55 (35–78)	LU (7A, 13B, 3HER2), TP (6)	dedicated for breast	RECIST 1.1, PERCIST 1.0	YES

Abbreviations: ∆: percentage change; 1D: unidimensional diameter; DC: ductal carcinoma; IDC: invasive ductal carcinoma; HER2: Human Epidermal Growth Factor Receptor 2; ILC: invasive lobar carcinoma; LC: lobar carcinoma; LD: longest tumor diameter; TV: tumor volume; LU-A, B: Luminal-A, Luminal-B; MTV: metabolic tumor volume; MUC: mucinous; PERCIST: PET Response Criteria in Solid Tumors; RECIST: Response Evaluation Criteria in Solid Tumors; SUL: standardized uptake value corrected for lean body mass; SUV: standardized uptake value; TP: triple-negative; NR: not reported; pCR: pathological complete response.

**Table 3:** Summary of diagnostic performance of MRI and PET/CT to predict pCR.

Authors [Ref.]	Performance Measure	MRI	PET/CT	MRI + PET
	SE	69.6	SUVmax 100	NR
Amioka et al. [28]	SP	85.0	SUVmax 52.5	NR
	Acc	79.4	SUVmax 69.8	NR
	SE	ΔLD 66.67 ΔTV 66.67 ΔPE 66.67 ΔLD + ΔTV + ΔPE 66.67 ΔADC 66.67	ΔSUV 66.67	LD + SUV 33.33 TV + SUV 33.33 PE + SUV 33.33 ADC + SUV 33.33
An et al. [27]	SP	ΔLD 94.12 ΔTV 94.12 ΔPE 70.59 ΔLD + ΔTV + ΔPE 94.12 ΔADC 70.59	ΔSUV 92.31	LD + SUV 100 TV + SUV 100 PE + SUV 92.32 ADC + SUV 100
	Acc	ΔLD 90.00 ΔTV 90.00 ΔPE 70.00 ΔLD + ΔTV + ΔPE 90.00 ΔADC 70.00	ΔSUV 87.50	LD + SUV 87.50 TV + SUV 87.50 PE + SUV 81.25 ADC + SUV 87.50
Baysal et al. [22]	SE	86.96	PERCIST 100	NR
	SP	30.7	PERCIST 75.6	NR
	Acc	57.1	PERCIST 81.8	NR
Choi et al. [30]	SE	1D 88.2	SULpeak 100	NR
	SP	1D 62.5	SULpeak 25	NR
	Acc	1D 75.7	SULpeak 63.6	NR
Kim et al. [24]	SE	Δ diameter 64.7 Δ volume 91.2	ΔSUV 91.3	NR
	SP	Δ diameter 95.5 Δ volume 77.3	ΔSUV 73.3	NR
	Acc	Δ diameter 76.8 Δ volume 85.7	ΔSUV 81.6	NR
Kitajima et al. [20]	SE	RECIST1.1 28.6	PERCIST 100	NR
	SP	RECIST1.1 94.4	PERCIST 22.2	NR
	Acc	RECIST1.1 65.6	PERCIST 56.3	NR
Cho et al. [29]	SE	MRS 75.9	SUVmax 100 SUVpeak 100 TLG 79.3	NR
	SP	MRS 100	SUVmax 66.7 SUVpeak 66.7 TLG 100	NR
	Acc	MRS 91.1	SUVmax 82.2 SUVpeak 86.2 TLG 87.9	NR
Pahk et al. [26]	SE	Δ size 64.3	ΔSUV 85.7	NR
	SP	Δ size 71.4	ΔSUV 100	NR
	Acc	Δ size 65	ΔSUV 90	NR
Park et al. [18]	SE	DWI 100	SUV 100	DWI + SUV 100
	SP	DWI 70.4	SUV 77.8	DWI + SUV 88.9
	Acc	DWI 76.5	SUV 82.4	DWI + SUV 91.2
Pengel et al. [25]	SE	NR	NR	NR
	SP	NR	NR	NR
	Acc	NR	NR	NR
Schmitz et al. [19]	SE	ER+ 36.2 TP 45.5	NR	HER2+ 55.8
	SP	NR	NR	NR
	Acc	NR	NR	NR
Simo et al. [23]	SE	NR	NR	NR
	SP	NR	NR	NR
	Acc	NR	NR	NR
Tateishi et al. [17]	SE	Δ rate costant 51.7	ΔSUVmax 66.7	NR
	SP	Δ rate costant 92	ΔSUVmax 96.4	NR
	Acc	Δ rate costant 83.8	ΔSUVmax 90.1	NR
Tokuda et al. [21]	SE	100	dbPET 85.7 WB-PET 71.4	NR
	SP	50	dbPET 72.7 WB-PET 77.3	NR
	Acc	77.3	dbPET 82 WB-PET 73	NR

Abbreviations: ∆: percentage change; 1D: unidimensional diameter; ADC: apparent diffusion coefficient; dbPET: dedicated breast positron emission tomography; DWI: diffusion-weighted imaging; ER+: estrogen receptor; HER2: human epidermal growth factor receptor 2; LD: longest tumor diameter; MRS: magnetic resonance spectroscopy; NR: not reported; PE: contrast peak enhancement; PERCIST: PET Response Criteria in Solid Tumors; RECIST: Response Evaluation Criteria in Solid Tumors; SUL: standardized uptake value corrected for lean body mass; SUV: standardized uptake value; TLG: total lesion glycolysis; TP: triple-negative; TV: tumor volume; WB: whole-body.

**Table 4:** Summary of quality evaluation according to QUADAS-2 tool. Studies are classified as low, high, or unclear risk of bias or applicability concerns.

Study	Riks of Bias				Applicability Concerns		
	Patient Selection	Index Text	Reference Standard	Flow and Timing	Patient Selection	Index Test	Reference Standard
Tateishi; 2012 [17]	?	?	+	?	−	−	+
Park; 2012 [18]	?	?	+	?	+	+	+
Simo; 2013 [23]	+	?	+	?	+	−	+
Kim; 2014 [24]	+	?	+	?	+	+	+
Pengel; 2014 [25]	?	?	+	+	+	+	+
Pahk; 2015 [26]	−	?	+	+	−	+	+
An; 2015 [27]	?	+	+	+	+	+	+
Cho; 2016 [29]	?	?	+	+	+	+	+
Amioka; 2016 [28]	?	?	+	?	−	−	+
Choi; 2017 [30]	−	+	+	−	+	+	+
Schmitz; 2017 [19]	+	+	+	?	−	+	+
Kitajima; 2018 [20]	+	?	+	?	+	+	+
Tokuda; 2021 [21]	+	?	+	?	+	−	+
Baysal; 2022 [22]	?	?	+	?	+	+	+

+: low risk, −: high risk, ?: unclear risk.

## Data Availability

Data sharing not applicable.

## References

[1] Siegel R.L., Miller K.D., Fuchs H.E., Jemal A. (2022). Cancer statistics, 2022. CA Cancer J. Clin..

[2] Korde L.A., Somerfield M.R., Carey L.A., Crews J.R., Denduluri N., Hwang E.S., Khan S.A., Loibl S., Morris E.A., Perez A. (2021). Neoadjuvant Chemotherapy, Endocrine Therapy, and Targeted Therapy for Breast Cancer: ASCO Guideline. J. Clin. Oncol..

[3] Cardoso F., Kyriakides S., Ohno S., Penault-Llorca F., Poortmans P., Rubio I.T., Zackrisson S., Senkus E. (2019). ESMO Guidelines Committee. Early breast cancer: ESMO Clinical Practice Guidelines for diagnosis, treatment and follow-up. Ann. Oncol..

[4] Spring L.M., Bar Y., Isakoff S.J. (2022). The Evolving Role of Neoadjuvant Therapy for Operable Breast Cancer. J. Natl. Compr. Cancer Netw..

[5] Laas E., Labrosse J., Hamy A.S., Benchimol G., de Croze D., Feron J.G., Coussy F., Balezeau T., Guerin J., Lae M. (2021). Determination of breast cancer prognosis after neoadjuvant chemotherapy: Comparison of Residual Cancer Burden (RCB) and Neo-Bioscore. Br. J. Cancer.

[6] Volders J.H., Negenborn V.L., Spronk P.E., Krekel N.M.A., Schoonmade L.J., Meijer S., Rubio I.T., van den Tol M.P. (2018). Breast-conserving surgery following neoadjuvant therapy-a systematic review on surgical outcomes. Breast Cancer Res. Treat..

[7] Cortazar P., Zhang L., Untch M., Mehta K., Costantino J.P., Wolmark N., Bonnefoi H., Cameron D., Gianni L., Valagussa P. (2014). Pathological complete response and long-term clinical benefit in breast cancer: The CTNeoBC pooled analysis. Lancet.

[8] Von Minckwitz G., Untch M., Blohmer J.U., Costa S.D., Eidtmann H., Fasching P.A., Gerber B., Eiermann W., Hilfrich J., Huober J. (2012). Definition and impact of pathologic complete response on prognosis after neoadjuvant chemotherapy in various intrinsic breast cancer subtypes. J. Clin. Oncol..

[9] Brackstone M., Palma D., Tuck A.B., Scott L., Potvin K., Vandenberg T., Perera F., D’Souza D., Taves D., Kornecki A. (2017). Concurrent Neoadjuvant Chemotherapy and Radiation Therapy in Locally Advanced Breast Cancer. Int. J. Radiat. Oncol. Biol. Phys..

[10] Dialani V., Chadashvili T., Slanetz P.J. (2015). Role of Imaging in Neoadjuvant Therapy for Breast Cancer. Ann. Surg. Oncol..

[11] Reig B., Lewin A.A., Du L., Heacock L., Toth H.K., Heller S.L., Gao Y., Moy L. (2021). Breast mri for evaluation of response to neoadjuvant therapy. Radiographics.

[12] Negrão E.M.S., Bitencourt A.G.V., de Souza J.A., Marques E.F. (2019). Accuracy of breast magnetic resonance imaging in evaluating the response to neoadjuvant chemotherapy: A study of 310 cases at a cancer center. Radiol. Bras..

[13] Loo C.E., Straver M.E., Rodenhuis S., Muller S.H., Wesseling J., Vrancken Peeters M.J., Gilhuijs K.G. (2011). Magnetic resonance imaging response monitoring of breast cancer during neoadjuvant chemotherapy: Relevance of Breast Cancer Subtype. J. Clin. Oncol..

[14] Wahl R.L., Zasadny K., Helvie M., Hutchins G.D., Weber B., Cody R. (1993). Metabolic monitoring of breast cancer chemohormonotherapy using positron emission tomography: Initial evaluation. J. Clin. Oncol..

[15] McInnes M.D.F., Moher D., Thombs B.D., McGrath T.A., Bossuyt P.M., The PRISMA-DTA Group (2020). Preferred reporting items for systematic review and meta-analysis of diagnostic test accuracy studies (PRISMA-DTA): Explanation, elaboration, and checklist. BMJ.

[16] Whiting P.F., Rutjes A.W., Westwood M.E., Mallett S., Deeks J.J., Reitsma J.B., Leeflang M.M., Sterne J.A., Bossuyt P.M., QUADAS-2 Group (2011). QUADAS-2: A revised tool for the quality assessment of diagnostic accuracy studies. Ann. Intern. Med..

[17] Tateishi U., Miyake M., Nagaoka T., Terauchi T., Kubota K., Kinoshita T., Daisaki H., Macapinlac H.A. (2012). Neoadjuvant chemotherapy in breast cancer: Prediction of pathologic response with PET/CT and dynamic contrast-enhanced MR imaging—Prospective assessment. Radiology.

[18] Park S.H., Moon W.K., Cho N., Chang J.M., Im S.A., Park I.A., Kang K.W., Han W., Noh D.Y. (2012). Comparison of diffusion-weighted MR imaging and FDG PET/CT to predict pathological complete response to neoadjuvant chemotherapy in patients with breast cancer. Eur. Radiol..

[19] Schmitz A.M.T., Teixeira S.C., Pengel K.E., Loo C.E., Vogel W.V., Wesseling J., Rutgers E.J.T., Valdés Olmos R.A., Sonke G.S., Rodenhuis S. (2017). Monitoring tumor response to neoadjuvant chemotherapy using MRI & 18F-FDG PET/CT in breast cancer subtypes. PLoS ONE.

[20] Kitajima K., Miyoshi Y., Yamano T., Odawara S., Higuchi T., Yamakado K. (2018). Assessment of tumor response to neoadjuvant chemotherapy in patients with breast cancer using MRI and FDG-PET/CT-RECIST 1.1 vs. PERCIST 1.0. Med. Sci..

[21] Tokuda Y., Yanagawa M., Fujita Y., Honma K., Tanei T., Shimoda M., Miyake T., Naoi Y., Kim S.J., Shimazu K. (2021). Prediction of pathological complete response after neoadjuvant chemotherapy in breast cancer: Comparison of diagnostic performances of dedicated breast PET, whole-body PET, and dynamic contrast-enhanced MRI. Breast Cancer Res. Treat..

[22] Baysal H., Serdaroglu A.Y., Ozemir I.A., Baysal B., Gungor S., Erol C.I., Ozsoy M.S., Ekinci O., Alimoglu O. (2022). Comparison of Magnetic Resonance Imaging with Positron Emission Tomography/Computed Tomography in the Evaluation of Response to Neoadjuvant Therapy of Breast Cancer. J. Surg. Res..

[23] Simo M., Gonzales Cao M., Ubeda B., Treserras F., Ara C., Brown J., Fabregas R., Baules S., Martinez A., Cubido M. (2013). Tumor response evaluation to neoadjuvant chemotherapy by functional imaging technologies. Eur. J. Nucl. Med. Mol. Imaging.

[24] Kim T., Kang D.K., An Y.S., Yim H., Jung Y.S., Kim K.S., Kang S.Y., Kim T.H. (2014). Utility of MRI and PET/CT after neoadjuvant chemotherapy in breast cancer patients: Correlation with pathological response grading system based on tumor cellularity. Acta Radiol..

[25] Pengel K.E., Koolen B.B., Loo C.E., Vogel W.V., Wesseling J., Lips E.H., Rutgers E.J., Valdés Olmos R.A., Vrancken Peeters M.J., Rodenhuis S. (2014). Combined use of 18F-FDG PET/CT and MRI for response monitoring of breast cancer during neoadjuvant chemotherapy. Eur. J. Nucl. Med. Mol. Imaging.

[26] Pahk K., Kim S., Choe J.G. (2015). Early prediction of pathological complete response in luminal B type neoadjuvant chemotherapy-treated breast cancer patients: Comparison between interim 18 F-FDG PET/CT and MRI. Nucl. Med. Commun..

[27] An Y.Y., Kim S.H., Kang B.J., Lee A.W. (2015). Treatment response evaluation of breast cancer after neoadjuvant chemotherapy and usefulness of the imaging parameters of MRI and PET/CT. J. Korean Med. Sci..

[28] Amioka A., Masumoto N., Gouda N., Kajitani K., Shigematsu H., Emi A., Kadoya T., Okada M. (2016). Ability of contrast-enhanced ultrasonography to determine clinical responses of breast cancer to neoadjuvant chemotherapy. Jpn. J. Clin. Oncol..

[29] Cho N., Im S.A., Kang K.W., Park I.A., Song I.C., Lee K.H., Kim T.Y., Lee H., Chun I.K., Yoon H.J. (2016). Early prediction of response to neoadjuvant chemotherapy in breast cancer patients: Comparison of single-voxel 1H-magnetic resonance spectroscopy and 18F-fluorodeoxyglucose positron emission tomography. Eur. Radiol..

[30] Choi E.K., Yoo I.R., Kim S.H., Park S.Y., Hyun O.J., Kang B.J. (2018). The value of pre- and post-neoadjuvant chemotherapy F-18 FDG PET/CT scans in breast cancer: Comparison with MRI. Acta Radiol..

[31] O’Connor M.K., Tran T.D., Swanson T.N., Ellingson L.R., Hunt K.N., Whaley D.H. (2017). Improved visualization of breast tissue on a dedicated breast PET system through ergonomic redesign of the imaging table. EJNMMI Res..

[32] Mamounas E.P. (2015). Impact of Neoadjuvant Chemotherapy on Locoregional Surgical Treatment of Breast Cancer. Ann. Surg. Oncol..

[33] Gu Y.L., Pan S.M., Ren J., Yang Z.X., Jiang G.Q. (2017). Role of Magnetic Resonance Imaging in Detection of Pathologic Complete Remission in Breast Cancer Patients Treated with Neoadjuvant Chemotherapy: A Meta-analysis. Clin. Breast Cancer.

[34] Rauch G.M., Adrada B.E., Kuerer H.M., van la Parra R.F., Leung J.W., Yang W.T. (2017). Multimodality imaging for evaluating response to neoadjuvant chemotherapy in breast cancer. Am. J. Roentgenol..

[35] Scheel J.R., Kim E., Partridge S.C., Lehman C.D., Rosen M.A., Bernreuter W.K., Pisano E.D., Marques H.S., Morris E.A., Weatherall P.T. (2018). MRI, Clinical Examination, and Mammography for Preoperative Assessment of Residual Disease and Pathologic Complete Response after Neoadjuvant Chemotherapy for Breast Cancer: ACRIN 6657 Trial. AJR Am. J. Roentgenol..

[36] Gampenrieder S.P., Peer A., Weismann C., Meissnitzer M., Rinnerthaler G., Webhofer J., Westphal T., Riedmann M., Meissnitzer T., Egger H. (2019). Radiologic complete response (rCR) in contrast-enhanced magnetic resonance imaging (CE-MRI) after neoadjuvant chemotherapy for early breast cancer predicts recurrence-free survival but not pathologic complete response (pCR). Breast Cancer Res..

[37] Schrading S., Kuhl C.K. (2015). Breast Cancer: Influence of Taxanes on Response Assessment with Dynamic Contrast-enhanced MR Imaging. Radiology.

[38] You S., Kang D.K., Jung Y.S., An Y.S., Jeon G.S., Kim T.H. (2015). Evaluation of lymph node status after neoadjuvant chemotherapy in breast cancer patients: Comparison of diagnostic performance of ultrasound, MRI and ¹⁸F-FDG PET/CT. Br. J. Radiol..

[39] Hayashi N., Tsunoda H., Namura M., Ochi T., Suzuki K., Yamauchi H., Nakamura S. (2019). Magnetic Resonance Imaging Combined With Second-look Ultrasonography in Predicting Pathologic Complete Response After Neoadjuvant Chemotherapy in Primary Breast Cancer Patients. Clin. Breast Cancer.

[40] Wang Y., Zhang C., Liu J., Huang G. (2012). Is 18F-FDG PET accurate to predict neoadjuvant therapy response in breast cancer? A meta-analysis. Breast Cancer Res. Treat..

[41] Goktas Aydin S., Bilici A., Olmez O.F., Oven B.B., Acikgoz O., Cakir T., Basim P., Cakir A., Kutlu Y., Hamdard J. (2022). The Role of 18F-FDG PET/CT in Predicting the Neoadjuvant Treatment Response in Patients with Locally Advanced Breast Cancer. Breast Care.

[42] Li H., Yao L., Jin P., Hu L., Li X., Guo T., Yang K. (2018). MRI and PET/CT for evaluation of the pathological response to neoadjuvant chemotherapy in breast cancer: A systematic review and meta-analysis. Breast.

[43] Fowler A.M., Strigel R.M. (2022). Clinical advances in PET–MRI for breast cancer. Lancet Oncol..

[44] Roy S., Whitehead T.D., Li S., Ademuyiwa F.O., Wahl R.L., Dehdashti F., Shoghi K.I. (2022). Co-clinical FDG-PET radiomic signature in predicting response to neoadjuvant chemotherapy in triple-negative breast cancer. Eur. J. Nucl. Med. Mol. Imaging.

[45] Sekine C., Uchiyama N., Watase C., Murata T., Shiino S., Jimbo K., Iwamoto E., Takayama S., Kurihara H., Satomi K. (2022). Preliminary experiences of PET/MRI in predicting complete response in patients with breast cancer treated with neoadjuvant chemotherapy. Mol. Clin. Oncol..

[46] de Mooij C.M., van Nijnatten T.J.A., Goorts B., Kooreman L.F., Raymakers I.W.M., van Meijl S.P.L., de Boer M., Keymeule K.B.M.I., Wildberger J.E., Mottaghy F.M. (2023). Prediction of Primary tumor and Axillary Lymph Node Response to Neoadjuvant Chemo (Targeted) Therapy with with Dedicated Breast [18F]FDG PET/MRI in Breast Cancer. Cancers.

[47] Ming Y., Wu N., Qian T., Li X., Wan D.Q., Li C., Li Y., Wu Z., Wang X., Liu J. (2020). Progress and Future Trends in PET/CT and PET/MRI Molecular Imaging Approaches for Breast Cancer. Front. Oncol..

[48] Aertgeerts K., Levin I., Shi L., Snell G.P., Jennings A., Prasad G.S., Zhang Y., Kraus M.L., Salakian S., Sridhar V. (2005). Structural and kinetic analysis of the substrate specificity of human fibroblast activation protein α. J. Biol. Chem..

[49] Backhaus P., Burg M.C., Asmus I., Pixberg M., Büther F., Breyholz H.J., Yeh R., Weigel S.B., Stichling P., Heindel W. (2023). Initial Results of 68Ga-FAPI-46 PET/MRI to Assess Response to Neoadjuvant Chemotherapy in Breast Cancer. J. Nucl. Med..

[50] Evangelista L., Urso L., Caracciolo M., Stracuzzi F., Panareo S., Cistaro A., Catalano O. (2023). FDG PET/CT Volume-Based Quantitative Data and Survival Analysis in Breast Cancer Patients: A Systematic Review of the Literature. Curr. Med. Imaging.

[51] Evangelista L., Cervino A.R., Ghiotto C., Saibene T., Michieletto S., Fernando B., Orvieto E., Guarneri V., Conte P. (2015). Could semiquantitative FDG analysis add information to the prognosis in patients with stage II/III breast cancer undergoing neoadjuvant treatment?. Eur. J. Nucl. Med. Mol. Imaging.

[52] Urso L., Evangelista L., Alongi P., Quartuccio N., Cittanti C., Rambaldi I., Ortolan N., Borgia F., Nieri A., Uccelli L. (2022). The Value of Semiquantitative Parameters Derived from 18F-FDG PET/CT for Predicting Response to Neoadjuvant Chemotherapy in a Cohort of Patients with Different Molecular Subtypes of Breast Cancer. Cancers.

[53] Groheux D., Cochet A., Humbert O., Alberini J.L., Hindié E., Mankoff D. (2016). 18F-FDG PET/CT for staging and restaging of breast cancer. J. Nucl. Med..

[54] Urso L., Quartuccio N., Caracciolo M., Evangelista L., Schirone A., Frassoldati A., Arnone G., Panareo S., Bartolomei M. (2022). Impact on the long-term prognosis of FDG PET/CT in luminal-A and luminal-B breast cancer. Nucl. Med. Commun..

[55] Son S.H., Lee S.W., Jeong S.Y., Song B.I., Chae Y.S., Ahn B.C., Lee J. (2015). Whole-Body Metabolic Tumor Volume, as Determined by 18F-FDG PET/CT, as a Prognostic Factor of Outcome for Patients With Breast Cancer Who Have Distant Metastasis. Am. J. Roentgenol..

[56] Kitajima K., Miyoshi Y., Sekine T., Takei H., Ito K., Suto A., Kaida H., Ishii K., Daisaki H., Yamakado K. (2021). Harmonized pretreatment quantitative volume-based FDG-PET/CT parameters for prognosis of stage I-III breast cancer: Multicenter study. Oncotarget.

[57] Urso L., Manco L., Castello A., Evangelista L., Guidi G., Castellani M., Florimonte L., Cittanti C., Turra A., Panareo S. (2022). PET-Derived Radiomics and Artificial Intelligence in Breast Cancer: A Systematic Review. Int. J. Mol. Sci..

[58] Molina-García D., García-Vicente A.M., Pérez-Beteta J., Amo-Salas M., Martínez-González A., Tello-Galán M.J., Soriano-Castrejón Á., Pérez-García V.M. (2018). Intratumoral heterogeneity in 18F-FDG PET/CT by textural analysis in breast cancer as a predictive and prognostic subrogate. Ann. Nucl. Med..

[59] Umutlu L., Kirchner J., Bruckmann N.M., Morawitz J., Antoch G., Ting S., Bittner A.K., Hoffmann O., Häberle L., Ruckhäberle E. (2022). Multiparametric18F-FDG PET/MRI-Based Radiomics for Prediction of Pathological Complete Response to Neoadjuvant Chemotherapy in Breast Cancer. Cancers.

[60] Oliveira C., Oliveira F., Vaz S.C., Marques H.P., Cardoso F. (2023). Prediction of pathological response after neoadjuvant chemotherapy using baseline FDG PET heterogeneity features in breast cancer. Br. J. Radiol..

[61] Hustinx R., Pruim J., Lassmann M., Visvikis D. (2022). An EANM position paper on the application of artificial intelligence in nuclear medicine. Eur. J. Nucl. Med. Mol. Imaging.

